# Supervised Learning and Multi-Omics Integration Reveals Clinical Significance of Inner Membrane Mitochondrial Protein (IMMT) in Prognostic Prediction, Tumor Immune Microenvironment and Precision Medicine for Kidney Renal Clear Cell Carcinoma

**DOI:** 10.3390/ijms24108807

**Published:** 2023-05-15

**Authors:** Chun-Chi Chen, Pei-Yi Chu, Hung-Yu Lin

**Affiliations:** 1 Section of Urology, Departments of Surgery, Changhua Christian Hospital, Changhua 500, Taiwan; 63481@cch.org.tw; 2 Department of Post-Baccalaureate Medicine, College of Medicine, National Chung Hsing University, Taichung 402, Taiwan; 3 School of Medicine, College of Medicine, Fu Jen Catholic University, New Taipei City 242, Taiwan; 4 Department of Pathology, Show Chwan Memorial Hospital, Changhua 500, Taiwan; 5 National Institute of Cancer Research, National Health Research Institutes, Tainan 704, Taiwan; 6 Research Assistant Center, Show Chwan Memorial Hospital, Changhua 500, Taiwan

**Keywords:** kidney renal clear cell carcinoma, inner membrane mitochondrial protein, biomarker, supervised learning, prognosis, tumor immune microenvironment, precision medicine

## Abstract

Kidney renal clear cell carcinoma (KIRC) accounts for approximately 75% of all renal cancers. The prognosis for patients with metastatic KIRC is poor, with less than 10% surviving five years after diagnosis. Inner membrane mitochondrial protein (IMMT) plays a crucial role in shaping the inner mitochondrial membrane (IMM), regulation of metabolism and innate immunity. However, the clinical relevance of IMMT in KIRC is not yet fully understood, and its role in shaping the tumor immune microenvironment (TIME) remains unclear. This study aimed to investigate the clinical significance of IMMT in KIRC using a combination of supervised learning and multi-omics integration. The supervised learning principle was applied to analyze a TCGA dataset, which was downloaded and split into training and test datasets. The training dataset was used to train the prediction model, while the test and the entire TCGA dataset were used to evaluate its performance. Based on the risk score, the cutoff between the low and high IMMT group was set at median value. A Kaplan-Meier curve, receiver operating characteristic (ROC) curve, principal component analysis (PCA) and Spearman’s correlation were conducted to evaluate the prediction ability of the model. Gene Set Enrichment Analysis (GSEA) was used to investigate the critical biological pathways. Immunogenicity, immunological landscape and single-cell analysis were performed to examine the TIME. Databases including Gene Expression Omnibus (GEO), Human Protein Atlas (HPA) and Clinical Proteomic Tumor Analysis Consortium (CPTAC) were employed for inter-database verification. Pharmacogenetic prediction was analyzed via single-guide RNA (sgRNA)-based drug sensitivity screening using Q-omics v.1.30. Low expressions of IMMT in tumor predicted dismal prognosis in KIRC patients and correlated with KIRC progression. GSEA revealed that low expressions of IMMT were implicated in mitochondrial inhibition and angiogenetic activation. In addition, low IMMT expressions had associations with reduced immunogenicity and an immunosuppressive TIME. Inter-database verification corroborated the correlation between low IMMT expressions, KIRC tumors and the immunosuppressive TIME. Pharmacogenetic prediction identified lestaurtinib as a potent drug for KIRC in the context of low IMMT expressions. This study highlights the potential of IMMT as a novel biomarker, prognostic predictor and pharmacogenetic predictor to inform the development of more personalized and effective cancer treatments. Additionally, it provides important insights into the role of IMMT in the mechanism underlying mitochondrial activity and angiogenesis development in KIRC, which suggests IMMT as a promising target for the development of new therapies.

## 1. Introduction

Kidney cancer is a prevalent cancer worldwide, and it is among the top ten most common types of cancer. Its global incidence is estimated to be approximately 2% [1]. Unfortunately, for a long time, surgical intervention has been the only viable treatment option for kidney cancer patients, and their survival rates have rarely exceeded one year [2]. Renal cancer encompasses various histological subtypes, each with a unique molecular landscape [3]. The most prevalent subtype is kidney renal clear cell carcinoma (KIRC), which is an adenocarcinoma derived from renal tubular epithelial cells and accounts for approximately 75% of all renal cancers. However, the prognosis for patients with metastatic KIRC is poor, with less than 10% surviving the five years after diagnosis [3]. Currently, there are limited tools for the early diagnosis and accurate prognostic prediction of KIRC, which often leads to delayed treatment and poorer outcomes for patients. Therefore, there is a critical unmet medical need for the development of early and precise biomarkers for the diagnosis and treatment of KIRC.

The Inner membrane mitochondrial protein (IMMT), also known as mitofilin or Mic60, is an essential component of the mitochondrial contact site and cristae organizing system (MICOS) complex [4], which plays a crucial role in shaping the inner mitochondrial membrane (IMM). The proper maintenance of the inner membrane architecture, including the formation of cristae junctions, depends on the presence of IMMT [5]. Down-regulation of IMMT has been shown to induce the collapse of mitochondria integrity, loss of bioenergetics, oxidative damage, growth arrest and activation of pro-inflammatory signaling [6,7]. Recently, IMMT was identified as a promising biomarker for the diagnosis and prognosis of patients with breast cancer [8]. It was furthermore recognized as a potential therapeutic target for prostate adenocarcinoma [7] and breast cancer [8]. However, the clinical relevance of IMMT in KIRC is not yet fully understood, and its role in shaping the tumor immune microenvironment (TIME) remains unclear.

In this study, we aimed to investigate the clinical significance of IMMT in KIRC using a combination of supervised learning and multi-omics integration. By analyzing data from multiple sources, including gene expression and clinical outcome databases, we sought to identify the potential prognostic and therapeutic value of IMMT in KIRC. The results of this study could provide important insights into the development of new strategies for prognostic prediction and precision medicine in KIRC, ultimately improving patient outcomes and survival rates.

## 2. Results

### 2.1. Evaluation and Verification of Prognostic Significance of IMMT in KIRC

**Figure 1:**
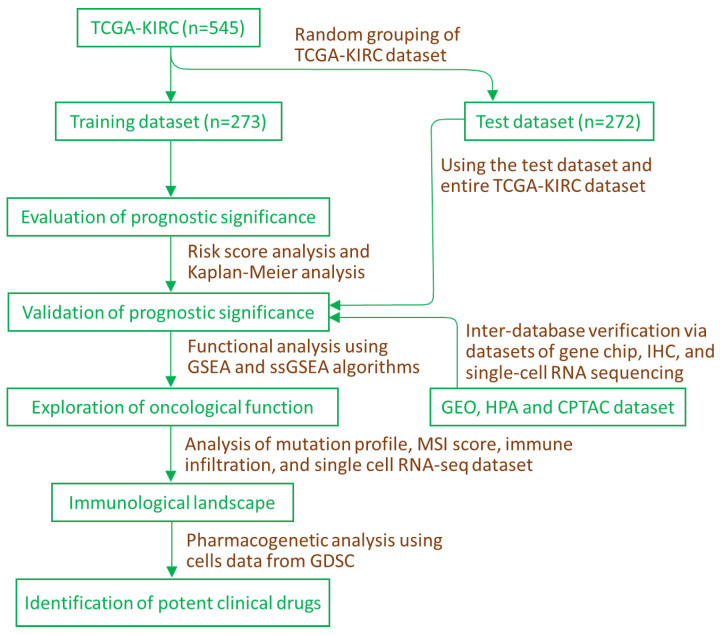
Workflow chart illustrating a supervised learning principle in conjunction with multi-omics analysis, inter-database verification and pharmacogenetic analysis. CPTAC, Clinical Proteomic Tumor Analysis Consortium. GDSC, Genomics of Drug Sensitivity in Cancer. GEO, Gene Expression Omnibus. HPA, human protein atlas. IHC, immunohistochemistry. GSEA, gene set enrichment analysis. KIRC, kidney renal clear cell carcinoma. ssGSEA, single-sample gene set enrichment analysis. TCGA, The Cancer Genomic Atlas.

**Figure 2:**
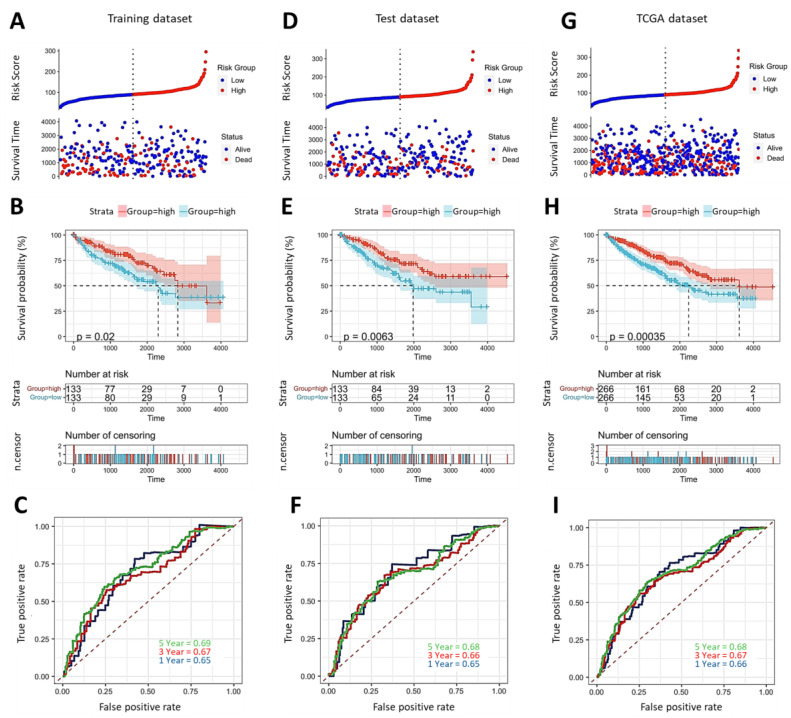
Low IMMT expression levels predict unfavorable prognoses. Risk score analysis of IMMT expressions with overall survival events in the training dataset (**A**), the test dataset (**D**) and the entire TCGA dataset (**G**). Overall survival probability of BC patients based on low/high IMMT grouping in the training dataset (**B**), the test dataset (**E**) and the entire TCGA dataset (**H**). Time-dependent ROC in the training dataset (**C**), the test dataset (**F**) and the entire TCGA dataset (**I**).

The workflow chart is depicted in Figure 1. Firstly, we split the KIRC data downloaded from the TCGA repository into a training dataset and a test dataset. Next, we employed risk score analysis to determine the cutoff point between low and high IMMT groups. As shown in Figure 2A, patients in the training group were divided into low and high levels using the median value. Kaplan-Meier analysis revealed that low IMMT expression was associated with a dismal overall survival rate (Figure 2B). The area under the curve (AUC) results of ROC for one-, three- and five-year overall survival prediction based on IMMT expression were 0.65, 0.67 and 0.69, respectively (Figure 2C), suggesting modest predictive capability. We subsequently estimated the accuracy of the prognostic significance using the test dataset and the entire TCGA dataset. In the test group, the cutoff based on the risk score (Figure 2D) exhibited a similar survival trend (Figure 2E) and ROC results (Figure 2F). Correspondingly, the prediction capability was validated in the entire TCGA dataset (Figure 2G–I). Together, these results indicate that low IMMT expression in tumors predicts a dismal prognosis in KIRC patients.

### 2.2. Low IMMT Expressions Correlate with KIRC Progression

**Figure 3:**
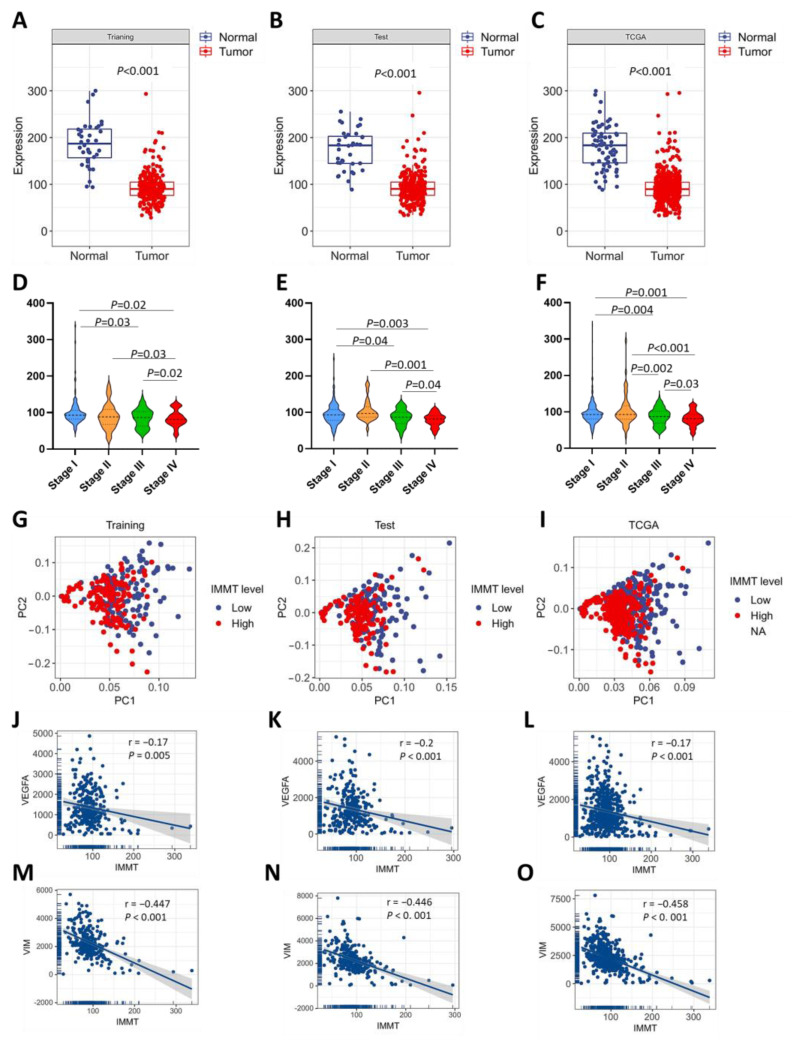
Low IMMT expressions are associated with KICR progression. Boxplot showing the IMMT expression levels in normal kidney tissue and KIRC tumor tissue in the training dataset (**A**), test dataset (**B**) and the entire dataset (**C**). Violin plot showing the IMMT expression levels in various tumor stages in the training dataset (**D**), test dataset (**E**) and the entire dataset (**F**). PCA plot of the training dataset (**G**), test dataset (**H**) and the entire dataset (**I**). Linear regression plot of Spearman’s correlation of IMMT with VEGFA expressions in the training dataset (**J**), test dataset (**K**) and the entire dataset (**L**). Linear regression plot of Spearman’s correlation of IMMT with VIM expressions in the training dataset (**M**), test dataset (**N**) and the entire dataset (**O**).

We next investigated the relationship between IMMT expression levels and disease progression. KIRC tumors presented lower expression levels of IMMT than normal kidney tissue in the training, test and entire TCAG dataset (Figure 3A–C). IMMT levels reduced as the KIRC stage progressed (Figure 3D–F). We performed principal component analysis (PCA) to explore the relationship between IMMT levels and the previously identified gene signatures of KIRC prognosis [9], including MMP9, MMP3, TWIST1, SNAI1, SNAI2, VIM, HIF1A, VEGFA, VEGFC, BIRC5 and TJP1. KIRC patients with low IMMT expressions shifted toward the upper right quarter of the PCA plot (Figure 3G–I). Moreover, the Spearman’s correlation results showed that IMMT had a negative correlation with VEGFA (Figure 3J–L) and VIM (Figure 3M–O). As a result, low IMMT expressions correlated with KIRC progression.

### 2.3. Implications of Low IMMT in Mitochondrial Inhibition and Angiogenetic Activation

**Figure 4:**
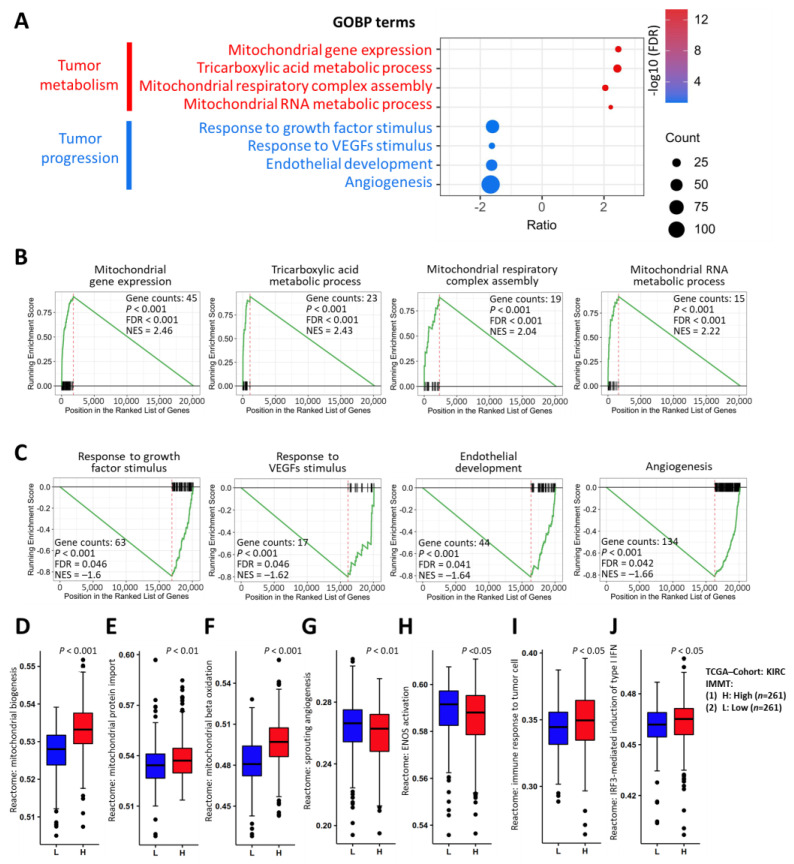
Functional analysis reveals the involvement of IMMT in mitochondrial activity and angiogenesis inhibition. Bubble plot illustrating the normalized enrichment score (NES) count of involved genes and -log10 false discovery rate value (FDR) of the Gene Ontology Biological Process (GOBP) terms (**A**). Gene set enrichment analysis (GSEA) plot showing the GOBP pathways in reference to mitochondrial metabolism (**B**) and growth/angiogenesis of tumors (**C**). Box plots showing the Reactome pathway score calculated using the single sample gene sets enrichment analysis (ssGSEA) method using the GSVA package, including mitochondrial biogenesis (**D**), mitochondrial protein import (**E**), mitochondrial beta oxidation (**F**), sprouting angiogenesis (**G**), endothelial nitric oxide synthase (ENOS) activation (**H**), immune response to tumor cell (**I**) and interferon regulatory factor 3 (IRF3)-mediated induction of type I interferon (IFN) (**J**).

To gain more insights into the underlying molecular basis of IMMT in KIRC, we utilized RNA sequencing data from TCGA to conduct gene set enrichment analysis (GSEA). As shown in Gene Ontology Biological Process (GOBP) terms (Figure 4A), IMMT in KIRC was involved in the activation of tumor metabolism (GOBP: mitochondrial gene expression, tricarboxylic acid metabolic process, mitochondrial respiratory complex assembly and mitochondrial RNA metabolic process) (Figure 4B) and in the inhibition of tumor progression (GOBP: response to growth factors stimulus, response to VEGFs stimulus, endothelial development and angiogenesis) (Figure 4C). Furthermore, Reactome pathways unveiled by ssGSEA confirmed the impacts of low IMMT expressions on the inhibition of mitochondrial biogenesis (Figure 4D), mitochondrial protein import (Figure 4E) and mitochondrial beta oxidation (Figure 4F), and on the promotion of sprouting angiogenesis (Figure 4G) and endothelial nitric oxide synthase (ENOS) activation (Figure 4H). Interestingly, we noted that low IMMT was associated with a suppressed immune response to tumor cells (Figure 4I) and interferon regulatory factor 3 (IRF3)-mediated induction of type I interferons (Figure 4J). Collectively, low expressions of IMMT were implicated in mitochondrial inhibition and angiogenetic activation.

### 2.4. Low IMMT Expressions Are Implicated in Reduced Immunogenicity and an Immunosuppressive TIME

**Figure 5:**
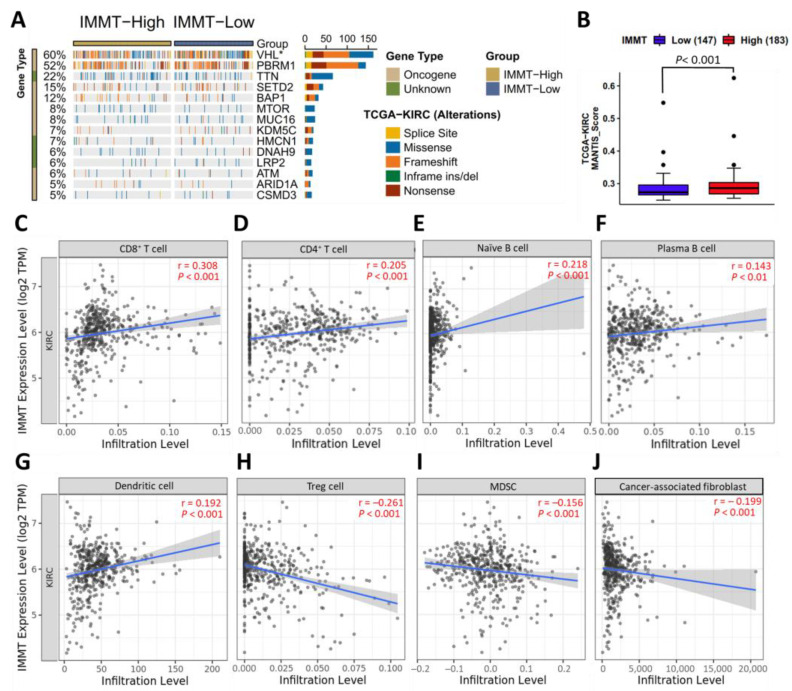
Implications of low IMMT expression in reduced immunogenicity and immunosuppressive TIME. Oncotplot exhibiting the mutation frequencies of the top 14 altered genes in KIRC patients harboring low and high expressions of IMMT. The differences in mutation frequencies between the low and high IMMT cohorts were statistically evaluated using Fisher’s exact tests (* *p* < 0.05) (**A**). Box plot of MANTIS scores in KIRC cohorts harboring low or high IMMT expressions (**B**). Linear regression plot representing the Spearman’s correlation results of IMMT with CD8+ T cells (**C**), CD4+ T cells (**D**), Naïve B cells (**E**), plasma B cells (**F**), dendritic cells (**G**), regulatory T (Treg) cells (**H**), myeloid-derived suppressor cells (MDSC) (**I**) and cancer-associated fibroblasts (**J**).

To explore the impact of IMMT expression on tumor immunogenicity, we assessed the mutation landscape and microsatellite analysis for normal tumor instability (MANTIS) score. MANTIS score is regarded as a predictor for the MSI status of tumors [10] and has been shown to positively correlate with immunogenicity. As shown in Figure 5A, low IMMT expression was associated with an increased mutation frequency of Von Hippel-Lindau Tumor Suppressor (VHL). The loss of VHL has been reported to activate hypoxia-inducible factors, which subsequently leads to VEGF-mediated angiogenesis in KIRC [11]. KIRC patients with low IMMT expression harbored lower MANTIS scores (Figure 5B). To more specifically clarify the immunological landscape, we looked into the relationship between IMMT and immune cell infiltration. We observed that IMMT levels had positive associations with immunoreactive cells, such as CD8+ T cells (Figure 5C), CD4+ T cells (Figure 5D), naïve B cells (Figure 5E), plasma B cells (Figure 5F) and dendritic cells (Figure 5G). In contrast, IMMT levels presented negative associations with immunosuppressive cells, including regulatory T (Treg) cells (Figure 5H), myeloid-derived suppressor cells (MDSC) (Figure 5I) and cancer-associated fibroblasts (Figure 5J). Taken together, low IMMT expressions are implicated in reduced immunogenicity and an immunosuppressive TIME.

### 2.5. Inter-Database Verification

**Figure 6:**
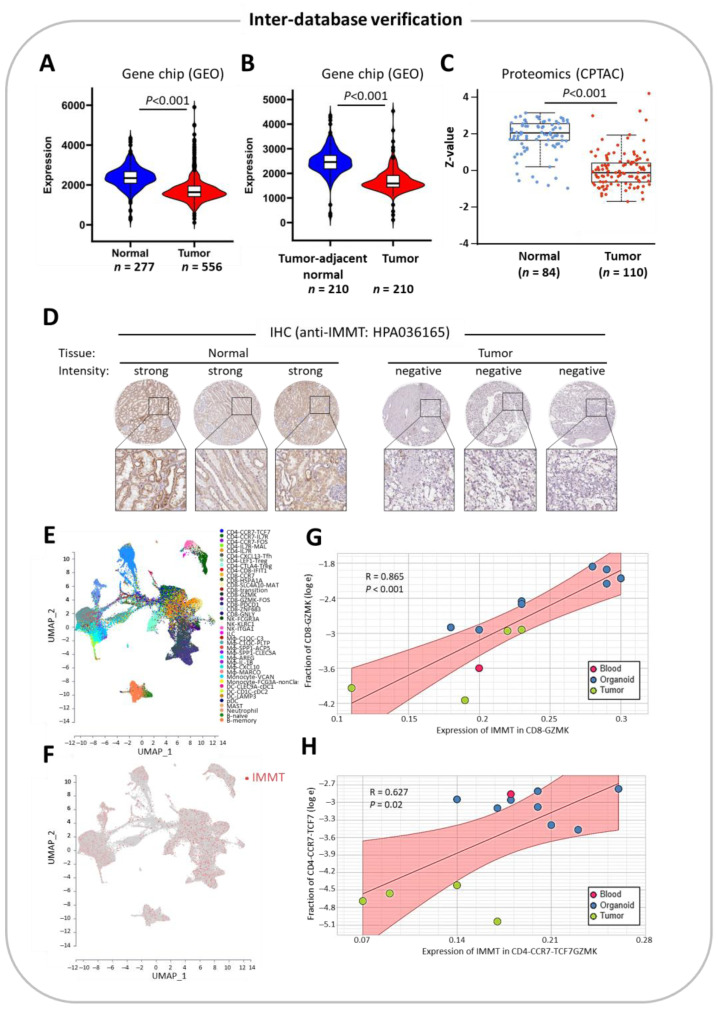
Inter-database verification confirms the clinical significance of IMMT in differential expression and immunological landscapes. Panels A and B show violin plots of gene chip-based expression levels of IMMT that were sourced from GEO datasets that display normal kidney tissue vs. KIRC tumor (**A**) and tumor-adjacent normal tissue vs. tumor tissue in KIRC samples (**B**). Box plot showing proteomics data of IMMT protein expression levels of normal kidney tissue and KIRC tumors, which were sourced from CPTAC datasets (**C**). IHC imaging of the HPA database showing the anti-IMMT (HPA036165)-detected IMMT protein expression levels of normal kidney tissue and KIRC tumors (**D**). Cell type deconvolution of GSE111360 single-cell RNA sequencing data. Panels E and F show Uniform Manifold Approximation and Projection (UMAP) plots showing all cell subtypes (**E**) and IMMT expression mapping onto cell types (**F**). Linear regression plot representing the Spearman’s correlation of IMMT expression levels in CD8+ cells expressing GZMK with the abundance of CD8+ cells expressing GZMK (**G**). Linear regression plot representing the Spearman’s correlation of IMMT expression levels in CD4+ cells expressing CCR7/TCF7 with the abundance of CD4+ cells expressing CCR7/TCF7 (**H**).

We further validated the clinical value of IMMT in the differential expression and prognostic prediction of KIRC using different databases. Transcriptomic data and proteomic data acquired from GEO and CPTAC, respectively (Figure 6A–C), confirmed the aforementioned findings based on the TCGA repository. Similarly, pathological inspections using IHC showed identical findings (Figure 6D). Cell type deconvolution using single-cell data from the GSE111360 dataset (Figure 6E) showed that the expression of IMMT was evenly distributed across various immune cells in the tumor microenvironment (Figure 6F). Furthermore, IMMT levels in CD8+ cells expressing GZMK and CD4+ cells expressing CCR7/TCF7 had positive correlations with their cellularity (Figure 6G,H). Therefore, inter-database verification corroborates the correlation between low IMMT expression with KIRC tumor and an immunosuppressive TIME.

### 2.6. Pharmacogenetic Prediction for Potent Drugs

**Figure 7:**
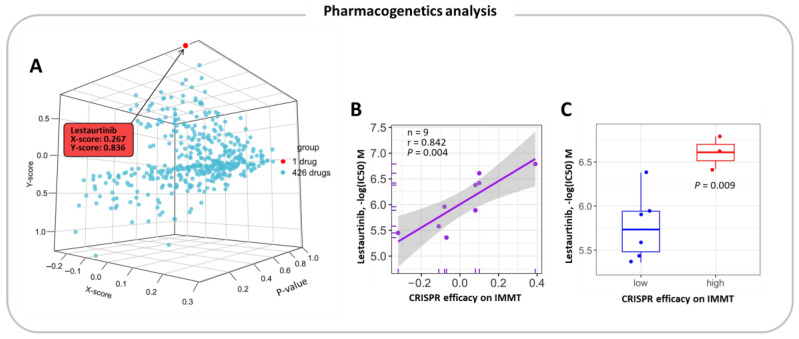
sgRNA-based pharmacogenetic prediction identifies lestaurtinib as a potent drug for KIRC cells with low IMMT expression. A three-dimensional scatter plot shows the cross-association between sgIMMT efficacy and response to 427 drugs (**A**). The X-score represents the log (fold change) of sgIMMT efficacy between samples of high and low response to the target drug. The Y-score represents the log (fold change) of targeted drug response between samples of high and low sgIMMT efficacy. The red dot represents one drug (lestaurtinib) out of the 427 drugs that showed statistically significant X- and Y-scores. The line regression plot shows the correlation between CRISPR efficacy on IMMT expression and cellular sensitivity (expressed as −log (IC50) M) to lestaurtinib (**B**). The box plots show comparisons of −log (IC50) M of lestaurtinib between KIRC cells with low (<50%) and high CRISPR efficacy (>50%) on IMMT expressions (**C**).

To investigate the potential of IMMT as a companion biomarker, we examined the association between CRISPR efficacy on IMMT and cellular response to 427 drugs in KIRC cell lines. We found that lestaurtinib exibited significantly altered cytotoxicity in response to CRISPR-perturbed IMMT gene expression (Figure 7A). In particular, the CRISPR efficacy on IMMT (i.e., the lower IMMT expressions) had a positive correlation with lestaurtinib toxicity in KIRC cells (Figure 7B). We then used a 50% CRISPR efficacy cutoff to divide cells into groups with low and high CRISPR efficacy on IMMT and compared lestaurtinib sensitivity. As shown in Figure 7C, cells with high CRISPR efficacy on IMMT presented greater lestaurtinib sensitivity. Overall, our pharmacogenetic prediction identifies lestaurtinib as a potent drug for KIRC in the context of low IMMT expression levels.

## 3. Discussion

**Figure 8:**
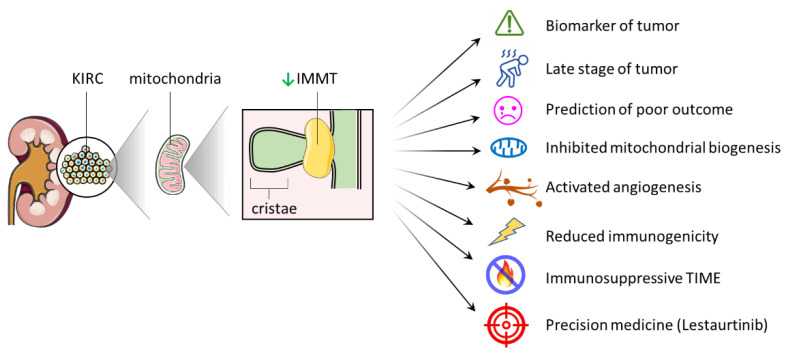
Graphic summary depicting the significance of IMMT in a variety of clinical scenarios and pathophysiological aspects of KIRC. Green arrow represents decreased IMMT expression.

The physiological role of IMMT in human diseases is an emerging area of research, with increasing attention being paid to its potential clinical relevance. Despite this, the precise clinical implications of IMMT expression in KIRC patients remain an unresolved issue, warranting further investigation. In this study, we employed a supervised learning model in conjunction with multi-omics analysis and inter-database verification to demonstrate the significance of IMMT as a novel biomarker, prognostic prediction, tumor progression, TIME shaping and precision medicine (Figure 8).

The findings of this study reveal that low expressions of IMMT in KIRC tumors are associated with a poor prognosis for patients. Furthermore, low IMMT expressions are correlated with KIRC progression, indicating a potential role for IMMT in tumor development. Our results also suggest that low expressions of IMMT are associated with mitochondrial inhibition and angiogenic activation, possibly contributing to the aggressive behavior of KIRC tumors. These findings are in line with observations in patients with pancreatic ductal adenocarcinoma [12]. On the contrary, low IMMT expression in patients with breast cancer indicates favorable survival outcomes compared to high IMMT expression [8]. In addition, low IMMT expression in breast cancer cells is involved in decreased mitochondrial activity, increased oxidative stress and suppressed cell cycle [8]. Further study is needed to clarify the exact mechanisms underlying this discrepancy.

Down-regulation of IMMT has been reported to be involved in local inflammation in kidneys with ischemia-reperfusion injuries. In breast cancer [8] and pancreatic ductal adenocarcinoma [12], low IMMT expression has been shown to be associated with an immunoreactive tumor microenvironment. Interestingly, our study demonstrates that low expression of IMMT is associated with reduced immunogenicity and an immunosuppressive TIME. However, the lack of experimental validation in vitro or in vivo poses a major limitation to gaining insight into the casual relationship between IMMT and immunoreactivity in KIRC. Further research is warranted to determine the exact mechanisms.

The inter-database verification strengthens the validity of the results and highlights the potential clinical implications of these findings. In particular, the results suggest that IMMT could serve as a useful biomarker for molecular diagnosis and clinicopathological inspections of KIRC tumors. Future research in this area may focus on developing more targeted therapies for KIRC patients based on the molecular diagnosis and identification of IMMT expression levels. Moreover, our pharmacogenetic prediction identifies lestaurtinib as a potent drug for KIRC in the context of low IMMT expressions. This finding suggests that personalized medicine approaches based on pharmacogenetics may be a useful tool for improving the efficacy of cancer treatments. Specifically, these results suggest that lestaurtinib may be a promising therapeutic option for patients with KIRC who have low IMMT expressions. Further research is needed to confirm these findings and explore the underlying mechanisms by which lestaurtinib interacts with IMMT expression. Overall, this study highlights the potential of IMMT as a novel biomarker, prognostic predictor and pharmacogenetic predictor to inform the development of more personalized and effective cancer treatments. Additionally, it provides important insights into the role of IMMT in the mechanism underlying mitochondrial activity and angiogenesis development in KIRC, suggesting IMMT as a promising target for the development of new therapies.

## 4. Materials and Methods

### 4.1. Data Sources and Supervised Learning Approach

RNA sequencing data retrieved from Genotype-Tissue Expression (GTEx) and the Cancer Genomic Atlas (TCGA) repository downloaded from OncoDB [13] were used to determine the differential expression of IMMT in normal kidney tissue and KIRC tumor tissue. A supervised learning-based principle was used to generate a prediction model in which we split the data into a training dataset and a test dataset. The TCGA dataset (*n* = 545) was randomly split to training (*n* = 273) and testing datasets (*n* = 272). The training dataset was used to train the prediction model, while the test and the entire TCGA dataset were used to evaluate its performance.

### 4.2. Analysis of Predictive Significance

We used principal component analysis (PCA) and Spearman’s correlation to analyze the association between IMMT and previously reported prognostic biomarkers for the KICR cohort [9]. Based on the risk score, the cutoff between the low and high IMMT groups was set at median value. The survival probability for groups harboring low and high IMMTs over time was estimated using Kaplan-Meier analysis. The prediction accuracy was evaluated using a receiver operating characteristic (ROC) curve. Immunohistochemistry (IHC) results from the Human Protein Atlas (HPA) [14,15,16] gene chip data were downloaded from Gene Expression Omnibus (GEO) using TNMplot [17], and proteomics data were downloaded from the Clinical Proteomic Tumor Analysis Consortium (CPTAC) using UALCAN [18,19].

### 4.3. Functional Enrichment Analysis

The initial step in analyzing the transcriptomics of the TCGA–KIRC cohort was to obtain the sample from LinkedOmics, a web portal that offers multi-omics data for 32 types of cancer and includes 11,158 TCGA project patients [20]. The HiSeq RNA platform was utilized to conduct the transcriptomics analysis. To investigate the association between IMMT expression and co-expressed genes, both positively and negatively correlated, the LinkFinder module was utilized. Additionally, Gene Set Enrichment Analysis (GSEA) was carried out using the WebGestalt tool in the LinkInterpreter module to identify enriched Gene Ontology Biological Process (GOBP) and miRNA targets [21,22]. For the GSEA, the rank criterion was set to the *p*-value, the gene size was set to five, and simulations were set at 500. This methodology allowed for a thorough analysis of the IMMT expression and its potential relationships with other genes and biological processes. For single-sample GSEA (ssGSEA), gene set scores were first calculated based on the rank of the member genes’ expression values in the entire gene expression matrix, and their significance was assessed using a permutation-based approach. Nest, the gene set scores were normalized to generate a final ssGSEA score for each gene set in the sample.

### 4.4. Immunogenicity, Immunological Landscape and Single-Cell Analysis

The mutation frequency and MANTIS score were accessed from cBioPortal to assess immunogenicity. To determine the immunological landscape, TIMER was used [23,24,25], which allows for the analysis of immune cell subtype infiltration based on the expression levels of a gene of interest. The scTIME Portal was used to analyze the transcriptomes of single cells in the tumor-immune microenvironment (TIME), using the GSE111360 dataset [26]. A UMAP plot of RNA sequencing of a human BC tumor using Smart-seq technology was generated.

### 4.5. Pharmacogenetic Prediction

The data on single-guide RNA (sgRNA)-based clustered regularly interspaced short palindromic repeats (CRISPR) screening for potent drugs were sourced from the Genomic Drug Sensitivity in Cancer (GDSC), Cancer Cell Line Encyclopedia (CCLE) and DeepMap datasets using Q-omics v.1.30 [27]. This software integrates and analyzes large-scale datasets of various types of molecular and biochemical information, such as genomics, transcriptomics, proteomics, and metabolomics, in order to gain insights into the complex biological processes that underlie various diseases and phenotypes.

### 4.6. Statistical Analysis

Statistical comparisons were conducted as previously described [28]. Briefly, unpaired t-tests and one-way analyses of variance (ANOVA) were employed to analyze quantitative data of two-group and three-or-more-group comparisons, respectively. The normal distribution of the IMMT expression data was examined using quantile–quantile (q–q) plot. The IMMT expression data of normal and tumor samples in the training dataset (Supplementary Figure S1A), test dataset (Supplementary Figure S1B) and entire TCGA dataset (Supplementary Figure S1C) showed straight lines, indicating normally distributed data. Spearman’s correlation coefficient was utilized to determine the relationship between two variables. The analysis involved the calculation of *p*-values for overall survival using the log-rank test. A comparison of the mutation frequencies between groups was made using the Fisher’s exact test.

## Data Availability

The data supporting the conclusions of this article are included within the article.

## Supplementary Materials

The supporting information can be downloaded at: https://www.mdpi.com/article/10.3390/ijms24108807/s1.
