# Supplementary material for: Supervised Learning and Multi-Omics Integration Reveals Clinical Significance of Inner Membrane Mitochondrial Protein (IMMT) in Prognostic Prediction, Tumor Immune Microenvironment and Precision Medicine for Kidney Renal Clear Cell Carcinoma
Source: Int J Mol Sci. 2023 May 15;24(10):8807. doi: 10.3390/ijms24108807 (PMC10218256; doi:10.3390/ijms24108807)
Supplement: Supplementary file 1 [file ijms-24-08807-s001.zip › ijms-2371471-supplementary.pdf]

Supplementary Materials

Supervised Learning and Multi-omics Integration Reveals Clinical Significance of Inner Membrane Mitochondrial Protein (IMMT) in Prognostic Prediction, Tumor Immune Microenvironment and Precision Medicine for Kidney Renal Clear Cell Carcinoma

Chun-Chi Chen [1], Pei-Yi Chu [2,3,4,5,*] and Hung-Yu Lin [2,6,*]

[1] Section of Urology, Departments of Surgery, Changhua Christian Hospital, Changhua 500, Taiwan; 63481@cch.org.tw
[2] Department of Post-Baccalaureate Medicine, College of Medicine, National Chung Hsing University, Taichung 402, Taiwan
[3] School of Medicine, College of Medicine, Fu Jen Catholic University, New Taipei City 242, Taiwan
[4] Department of Pathology, Show Chwan Memorial Hospital, Changhua 500, Taiwan
[5] National Institute of Cancer Research, National Health Research Institutes, Tainan 704, Taiwan
[6] Research Assistant Center, Show Chwan Memorial Hospital, Changhua 500, Taiwan
* Correspondence: chu.peiyi@msa.hinet.net (P.-Y.C.); linhungyu700218@gmail.com (H.-Y.L.)

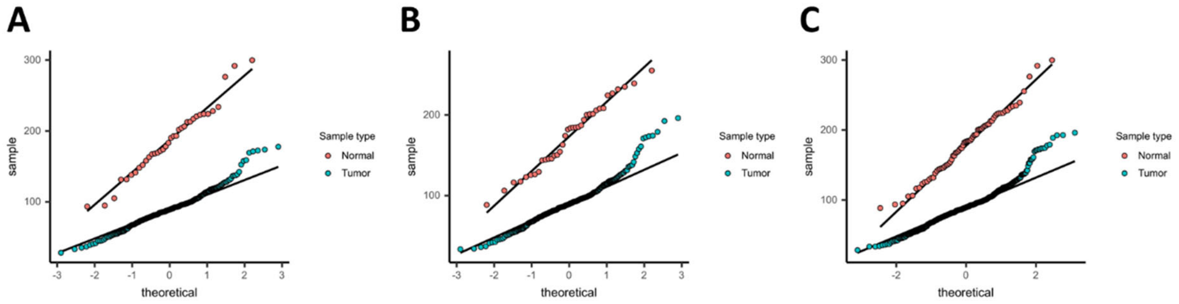

Supplementary Figure S1. Quantile-quantile (q-q) plot illustrating the distribution of IMMT expression levels of normal and tumor data in Training (A), Test and (B) entire TCGA dataset (C).
